# Clinical relevance of *KRAS* mutation detection in metastatic colorectal cancer treated by Cetuximab plus chemotherapy

**DOI:** 10.1038/sj.bjc.6603685

**Published:** 2007-03-20

**Authors:** F Di Fiore, F Blanchard, F Charbonnier, F Le Pessot, A Lamy, M P Galais, L Bastit, A Killian, R Sesboüé, J J Tuech, A M Queuniet, B Paillot, J C Sabourin, F Michot, P Michel, T Frebourg

**Affiliations:** 1 Digestive Oncology Unit, Department of Hepato-Gastroenterology, Rouen University Hospital, Northwest Canceropole, France and Inserm U614, Faculty of Medicine, Northwest Canceropole, Rouen, France; 2 Inserm U614, Faculty of Medicine, Northwest Canceropole, Rouen, France; 3 Department of Pathology, Rouen University Hospital, Northwest Canceropole, France; 4 Digestive Oncology Unit, Department of Hepato-Gastroenterology, Caen University Hospital and Francois Baclesse Centre, Caen, Northwest Canceropole, France; 5 Oncology Unit, St Hilaire Medical Centre, Rouen, France; 6 Department of Surgery, Rouen University Hospital, Northwest Canceropole, France; 7 Digestive Oncology Unit, Department of Hepato-Gastroenterology, Elbeuf Hospital, France

**Keywords:** colorectal cancer, EGFR, *KRAS*, molecular markers, mutation

## Abstract

The predictive value of *KRAS* mutation in metastatic colorectal cancer (MCRC) patients treated with cetuximab plus chemotherapy has recently been suggested. In our study, 59 patients with a chemotherapy-refractory MCRC treated with cetuximab plus chemotherapy were included and clinical response was evaluated according to response evaluation criteria in solid tumours (RECIST). Tumours were screened for *KRAS* mutations using first direct sequencing, then two sensitive methods based on SNaPshot and PCR-ligase chain reaction (LCR) assays. Clinical response was evaluated according to gene mutations using the Fisher exact test. Times to progression (TTP) were calculated using the Kaplan–Meier method and compared with log-rank test. A *KRAS* mutation was detected in 22 out of 59 tumours and, in six cases, was missed by sequencing analysis but detected using the SNaPshot and PCR-LCR assays. Remarkably, no *KRAS* mutation was found in the 12 patients with clinical response. *KRAS* mutation was associated with disease progression (*P*=0.0005) and TTP was significantly decreased in mutated *KRAS* patients (3 *vs* 5.5 months, *P*=0.015). Our study confirms that *KRAS* mutation is highly predictive of a non-response to cetuximab plus chemotherapy in MCRC and highlights the need to use sensitive molecular methods, such as SNaPshot or PCR-LCR assays, to ensure an efficient mutation detection.

Colorectal cancer is one of the most common tumours and a major cause of cancer death worldwide. The median overall survival of patients with a metastatic colorectal cancer (MCRC) has increased from 12 months to approximately 20 months in the past decade (Douillard *et al*, 2003; Meyerhardt and Mayer, 2005; Saunders and Iveson, 2006). This dramatic improvement was mainly due to the introduction of both active new chemotherapeutic agents and novel targeted drugs. The rationale of targeted therapies is to inhibit biological pathways and key molecules involved in tumour growth and progression. In CRC, the novel therapies that are currently used target the vascular endothelial growth factor and epidermal growth factor (EGF) signaling pathways (Kabbinavar *et al*, 2003; Hurwitz *et al*, 2004; Saltz *et al*, 2004). The variability of the MCRC clinical response to anti-EGFR agents has highlighted the urgent need to identify reliable markers with a predictive value to select the appropriate patients who can benefit from these treatments. Essential in this context are two recent studies (Moroni *et al*, 2005; Lievre *et al*, 2006) reporting the characterization of molecular markers predictive of anti-EGFR antibodies sensitivity in MCRC. The first study, performed in 31 MCRC patients, showed that an *EGFR* gene copy number increase was associated with a clinical response to anti-EGFR agents and that mutation of *KRAS*, downstream of EGF signaling, did not correlate with treatment sensitivity (Moroni *et al*, 2005). In contrast, the second study including 30 MCRC patients reported that *KRAS* mutation was highly predictive of tumour resistance to cetuximab (Lievre *et al*, 2006).

The aim of the present study was to determine the clinical relevance of *KRAS* mutation detection in MCRC patients treated with cetuximab.

## MATERIALS AND METHODS

### Patients

Patients with an MCRC treated with cetuximab (Erbitux®, Merck, Lyon, France) between April 2004 and December 2005 and for whom tumour DNA was available were included. All patients had previously received at least one chemotherapy regimen for MCRC. Cetuximab regimen was associated either with irinotecan or with oxaliplatin. Tumour response was evaluated according to the response evaluation criteria in solid tumours (Therasse *et al*, 2000). Patient's tumour response to cetuximab was classified as complete response (CR), partial response (PR), stable disease (SD) or progressive disease (PD).

### DNA extraction

DNA was extracted from paraffin-embedded tumour tissue samples using the DNA extraction kits from Takara (Madison, WI, USA) or Ambion (Huntingdon, Cambridgeshire, UK), according to the manufacturer's instructions. Among the 59 patients analysed, DNA was extracted from the primary tumour in 53 cases and from metastases in the six remaining cases.

### Sequencing analysis

*KRAS* exon 2 was PCR-amplified from tumour DNA using the following sense and antisense primers: 5′-AAGGCCTGCTGAAAATGACTG-3′ and 5′-CAAAGAATGGTCCTGCACCAG-3′. After purification using the gel extraction kit from Qiagen (Courtaboeuf, France), PCR products were sequenced using the Big Dye V3.1 Terminator Kit (Applied Biosystems, Foster City, CA, USA) and an ABI Prism 377 or 3100 DNA sequencer (Applied Biosystems). Considering the presence of non malignant cells in tumour samples, the presence of an heterozygous *KRAS* mutation in the tumour was defined as the appearance of a mutant peak with an height of at least one-third of that of the wild type. All sequencing analyses were performed at least twice on two independent PCRs.

### SNaPshot multiplex assay

After purification using gel extraction kit, PCR-amplified *KRAS* exon 2 was analysed for the presence of *KRAS* mutations at nucleotides c.34, c.35, c.37 and c.38, using the ABI PRISM SNaPshot Multiplex kit (Applied Biosystems, Foster City, CA, USA) and four primers including at their 5′ end, an additional tail allowing their simultaneous detection. The sequences of the sense primers allowing the extension at nucleotides c.34, c.35, c.37 and c.38 were, respectively, 5′-AACTTGTGGTAGTTGGAGCT-3′, 5′-N_10_ ACTTGTGGTAGTTGGAGCTG-3′, 5′-N_20_ TTGTGGTAGTTGGAGCTGGT-3′ and 5′-N_30_ TGTGGTAGTTGGAGCTGGTG-3′ (N indicating the additional nucleotides). The multiplex SNaPshot reaction was performed in a final volume of 10 *μ*l, containing one-fifth of the PCR reaction, 2.5 *μ*l of the SNaPshot Multiplex Ready Reaction Mix, 1 *μ*l of sequencing buffer from the Big Dye V3.1 Terminator Kit and SNaPshot primers at a concentration of 0.02–0.05 *μ*M. Cycling conditions were 25 cycles of rapid thermal ramp to 96°C, 96°C for 10 s; rapid thermal ramp to 50°C, 50°C for 5 s; and rapid thermal ramp to 60°C and 60°C for 30 s. SNaPshot products were then treated 1 h at 37°C with 3 U of shrimp alkaline phosphatase (Amersham Biosciences/GE Healthcare Europe GmbH, Saclay, France). After heat inactivation of the alkaline phosphatase 15 min at 75°C, labelled products were separated using a 25 min run on an ABI Prism 3130 DNA sequencer and data were analysed using the GeneMapper Analysis Software version 4.0 (Applied Biosystems).

### PCR-LCR

*KRAS* exon 2 was PCR-amplified using the sense primer 5′-AAGGTACTGGTGGAGTATTTGATAGTG-3′ and the antisense primer 5′-TGTTGGATCATATTCGTCCACAAAA-3′. Ligase chain reaction (LCR) was then performed, as described by Shi *et al* (2004), on PCR-amplified exon 2 of *KRAS*, after purification using the Qiagen gel extraction kit. The c.34 nucleotide was explored using the specific upstream primer 5′-AACTTGTGGTAGTTGGAGATA-3′ (c.34 G>A, p.G12S), or 5′-ACTTGTGGTAGTTGGAGATT-3′ (c.34 G>T, p.G12C) and the common downstream primer 5′-GTGGCGTAGGCAAGAGTGC-3′; the c.35 nucleotide using the specific upstream primer 5′-TTGTGGTAGTTGGAGCTGA-3′ (c.35 G>A; p.G12D), or 5′-GTTGTGGTAGTTGGAGCTGC-3′ (c.35 G>C; p.G12A), or 5′-TTGTGGTAGTTGGAGCTGT-3′ (c.35G>T; p.G12V) and the common downstream primer 5′-TGGCGTAGGCAAGAGTGCC-3′; the c.38 nucleotide using the specific upstream primer 5′-TGGTAGTTGGAGCTGGTGA-3′ (c.38 G>A; p.G13D) and the downstream primer 5′-CGTAGGCAAGAGTGCCTTGAC-3′. Upstream primers contain at their 5′ end the M13F additional sequence (5′-ACTGTAAAACGACGGCCAGTGT-3′) and downstream primers at their 3′ end the M13R additional sequence (5′-TGGTCATAGCTGTTTCCTGCA-3′). Upstream primers were 5′-6FAM labelled and the downstream primers were 5′ phosphorylated. Ligase chain reaction reactions were performed in a final volume of 12.5 *μ*l containing 2 U of *Pfu* DNA ligase (Stratagene, la Jolla, CA, USA), and 1.25 *μ*M of each primer. After denaturation at 95°C for 20 s, 40 two-steps cycles of 94°C for 10 s alternating with 65°C for 2 min were performed. Ligation products were analysed on an ABI Prism 3100 DNA sequencer and the Gene Scan V3.7.1 (Applied Biosystems). For each sample analysed, PCR-LCR was performed twice.

### Statistical analysis

Response to treatment according the mutational status was evaluated using the Fisher exact test. Patients with CR or PR or SD were considered as patients with controlled disease (CD). A *P*-value equal or <0.05 was considered to indicate statistical significance. The time to progression (TTP) was calculated as the period from the beginning of treatment to the first observation of disease progression or to death. The TTP were estimated using the Kaplan–Meier method and compared with the log-rank test.

## RESULTS

A total of 59 patients were assessed in the present study. After 3 months of treatment, 31 patients (52.5%) had a CD, 12 patients (20.3%) had a CR or PR (2 and 10 patients, respectively) and 19 (32.2%) had an SD. The TTP in patients with CD was 6 months, as compared to 3 months, in patients with PD (*P*<0.0001).

### *KRAS* mutation and response to treatment with cetuximab plus chemotherapy

**Figure 1:**
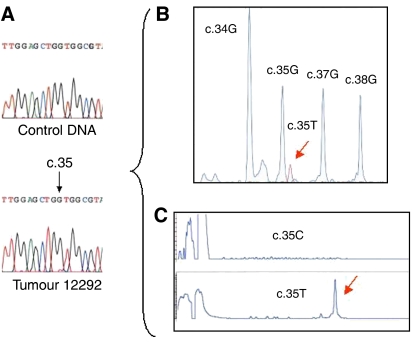
Detection by SNaPShot and PCR-LCR assays of *KRAS* mutations not detected by direct sequencing. Direct sequencing of *KRAS* exon 2 from control DNA and tumour 12292. The black arrow indicates the nucleotide c.35 (**A**). SNaPShot detection of the c.35G>T (p.G12V) mutation in tumour 12292. Each peak corresponds to a specific extended primer. The red arrows indicates the peak specific of the c.35G>T mutation (**B**). PCR-LCR analysis of tumour 12292, using a dye-labelled primer specific for the mutant c.35G>C (p.G12A) or c.35G>T (p.G12V) *KRAS* allele. The arrow indicates the peak specific of the c.35G>T mutation (**C**). Note that the c.35G>T mutation detected by both the SNaPShot and PCR-LCR assays cannot be clearly detected by sequencing analysis alone.

We detected a *KRAS* mutation by sequencing analysis of DNA extracted from tumour sample in 16 out of 59 (27%) patients (Table 1). Among the 16 patients harbouring a somatic *KRAS* mutation, 13 had a PD and three had an SD. Remarkably, no *KRAS* mutation was found in the 12 patients with CR or PR. Considering that the genetic heterogeneity of tumours may hamper the detection by direct sequencing of heterozygous mutations present in a small fraction of tumour cells, we screened the tumours without detectable *KRAS* mutations, using two sensitive methods able to detect specifically *KRAS* exon 2 mutations. We developed a multiplex SNaPshot assay based on primer extension able to detect simultaneously in a single tube the different *KRAS* mutations and a fluorescent PCR-LCR assay. These two analyses were performed in 11 out of 12 CR/PR patients, in 15 out of 16 patients with SD and in the 15 PD patients, in whom direct sequencing from tumour DNA had revealed no mutation. Five additional *KRAS* mutations were detected by both methods (Figure 1) and one mutation was detected only by PCR-LCR assay. These six additional mutations were found in two SD and four PD patients (Table 1). SNaPshot and PCR-LCR assays confirmed the absence of *KRAS* mutations in the CR/PR patients. In this series of 59 MCRC, sequencing analysis completed by SNaPshot multiplex and PCR-LCR assays led, therefore, to the detection of a *KRAS* mutation in 22 samples (37%). The presence of *KRAS* mutation was in this series significantly associated with PD (*P*=0.0005). The predictive value of *KRAS* mutation for PD could be estimated to 77.2%, and the sensitivity and specificity of *KRAS* mutation for progression to treatment (CD *vs* PD) to 60.7 and 83.8%, respectively. The TTP was significantly decreased in patients harbouring *KRAS* mutation as compared to those without detectable mutation (3 *vs* 5.5 months, *P*=0.015).

## DISCUSSION

This study performed on 59 MCRC patients confirms that the presence of *KRAS* mutation in tumour is highly predictive of a non-response to treatment based on cetuximab plus chemotherapy, as shown previously in a series of 30 patients (Lievre *et al*, 2006). It is important to highlight that, in our series, the proportions of CR/PR, SD and PD patients were 20.3, 32.2 and 47.5%, respectively, and this distribution is similar to that reported in the randomised cetuximab trial (Cunningham *et al*, 2004). The relationship between *KRAS* status and sensitivity to anti-EGFR monoclonal antibodies had not been found previously by Moroni *et al* (2005) in a series of 31 patients. This discrepancy might probably be explained, at least in part, by the limited number of patients in these latter series. Direct sequencing allowed us to detect a *KRAS* mutation in 16 out of 59 patients (27%) with MCRC, and among the 43 patients without detectable *KRAS* mutations, 15 presented a PD. We hypothesized that we missed some *KRAS* mutations by direct sequencing of tumour DNA, since malignant tumours are genetically heterogeneous. Furthermore, it is important to highlight that our study was based on paraffin-embedded tumours from which it is more difficult to obtain high-quality DNA. Using two independent sensitive methods, respectively, based on SNaPshot and PCR-LCR assays specifically designed to detect *KRAS* mutation, we detected additional mutations in two patients with SD, four with PD but none in 11 with CR/PR. This demonstrates the need to use highly sensitive molecular techniques to ensure detection in tumours of mutations conferring resistance to treatments. Considering the heterogeneity of tumour cells, sampling tissue is particularly important. We determined using SnaPshot assay the *KRAS* status in two to three different areas of three tumours including two with *KRAS* mutation. These analyses showed that the results did not differ according to the site of the analysis. The absence of *KRAS* mutation in 11 out of 28 patients with PD in our series is probably explained by the fact that *KRAS* mutation is not the only genetic alteration conferring resistance to anti-EGFR antibodies. Indeed somatic alterations hitting other downstream effectors of the EGFR transduction cascade, such as *RAF*, *MEK* or *ERK*, may have a similar effect.

In conclusion, these results should prompt further studies on larger MCRC series to definitely establish the clinical relevance of *KRAS* mutation detection in anti-EGFR antibodies based on chemotherapy. They also highlight the need to use sensitive molecular methods to detect mutations conferring resistance and the two assays presented in this study should facilitate the detection of *KRAS* mutations in CRC, on a routine basis. A major criticism that should be made to all studies on predictive markers of clinical response to anti-EGFR agents in MCRC, including ours, is that the clinical response is evaluated on the metastatic disease whereas the presence of the molecular marker is assessed from the primary tumour. In the present study we had the opportunity to compare the *KRAS* mutational status between primary tumour and metastases in five patients whom samples were available. In these five patients, SnaPshot assay had indicated that a *KRAS* mutation was present in one case and absent in the remaining five cases. For these five patients, analysis of the corresponding metastatic site showed that the *KRAS* mutation status was identical between the primary tumour and metastases. Considering the genetic evolution of metastases compared to primary tumour, we think nevertheless that it will be important in the future to screen directly metastases for the presence of alterations conferring either sensitivity or resistance to these targeted therapies.

## Figures and Tables

**Table 1:** *KRAS* mutations and response status to cetuximab-based chemotherapy in 59 MCRC patients

	**Controlled disease**			
	**Complete**	**Partial**	**Stable**	**Progressive**
	**response**	**response**	**disease**	**disease**
*KRAS mutation*				
Present	0	0	3 (5)	13 (17)
Absent	2 (2)	10 (10)	16 (14)	15 (11)

Numbers in brackets correspond to the corrected numbers of patients when sequencing analysis was completed by SNaPshot and PCR–LCR assays.
